# Supplementary material for: End Sequence Analysis Toolkit (ESAT) expands the extractable information from single-cell RNA-seq data
Source: Genome Res. 2016 Oct;26(10):1397–410. doi: 10.1101/gr.207902.116 (PMC5052061; doi:10.1101/gr.207902.116)
Supplement: Supplemental Material [file supp_gr.207902.116_Supp_Materials.tar.gz › ESAT-esat_v0.1/doc/html/classbroad_1_1core_1_1annotation_1_1_annotation_reader_factory.html]

ESAT: broad.core.annotation.AnnotationReaderFactory Class Reference


ESAT


- Main Page
- Related Pages
- Packages
- Classes
- Files

- Class List
- Class Index
- Class Hierarchy
- Class Members

All Classes Namespaces Files Functions Variables Enumerator Pages

- broad
- core
- annotation
- AnnotationReaderFactory

Static Public Member Functions |
List of all members

broad.core.annotation.AnnotationReaderFactory Class Reference

|  |  |
| --- | --- |
| Static Public Member Functions | |
| static AnnotationReader  <?extends GenomicAnnotation > | create (String sourceFile, String annotationType) throws ParseException, IOException |
|  | |
| static AnnotationReader  <?extends GenomicAnnotation > | create (String annotationType) throws ParseException, IOException |
|  | |
| static AnnotationReader  <?extends GenomicAnnotation > | create (String sourceFile, String annotationType, final double minScore) throws ParseException, IOException |
|  | |
| static AnnotationReader  <?extends GenomicAnnotation > | create (String sourceFile, String annotationType, final String chromosome) throws ParseException, IOException |
|  | |

## Member Function Documentation

|  |  |  |  |
| --- | --- | --- | --- |
| static AnnotationReader<? extends GenomicAnnotation> broad.core.annotation.AnnotationReaderFactory.create | ( | String | *sourceFile*, |
|  |  | String | *annotationType* |
|  | ) |  | throws ParseException, IOException |

static

Here is the caller graph for this function:

|  |  |  |  |  |  |
| --- | --- | --- | --- | --- | --- |
| static AnnotationReader<? extends GenomicAnnotation> broad.core.annotation.AnnotationReaderFactory.create | ( | String | *annotationType* | ) | throws ParseException, IOException |

static

|  |  |  |  |
| --- | --- | --- | --- |
| static AnnotationReader<? extends GenomicAnnotation> broad.core.annotation.AnnotationReaderFactory.create | ( | String | *sourceFile*, |
|  |  | String | *annotationType*, |
|  |  | final double | *minScore* |
|  | ) |  | throws ParseException, IOException |

static

|  |  |  |  |
| --- | --- | --- | --- |
| static AnnotationReader<? extends GenomicAnnotation> broad.core.annotation.AnnotationReaderFactory.create | ( | String | *sourceFile*, |
|  |  | String | *annotationType*, |
|  |  | final String | *chromosome* |
|  | ) |  | throws ParseException, IOException |

static

Here is the call graph for this function:

---

The documentation for this class was generated from the following file:

- java/broad/core/annotation/AnnotationReaderFactory.java


---

Generated on Fri Aug 15 2014 15:10:50 for ESAT by  

 1.8.7
